# Hormones regulate the flowering process in saffron differently depending on the developmental stage

**DOI:** 10.3389/fpls.2023.1107172

**Published:** 2023-03-09

**Authors:** Deepika Singh, Sahiba Sharma, Joel Jose-Santhi, Diksha Kalia, Rajesh Kumar Singh

**Affiliations:** ^1^ Biotechnology Division, Council of Scientific and Industrial Research (CSIR)-Institute of Himalayan Bioresource Technology, Palampur, HP, India; ^2^ Academy of Scientific and Innovative Research (AcSIR), Ghaziabad, India

**Keywords:** flowering (evocation), phytohormones, homeotic genes, floral integrators, floral induction

## Abstract

Flowering in saffron is a highly complex process regulated by the synchronized action of environmental cues and endogenous signals. Hormonal regulation of flowering is a very important process controlling flowering in several plants, but it has not been studied in saffron. Flowering in saffron is a continual process completed in months with distinct developmental phases, mainly divided into flowering induction and flower organogenesis/formation. In the present study, we investigated how phytohormones affect the flowering process at different developmental stages. The results suggest that different hormones differentially affect flower induction and formation in saffron. The exogenous treatment of flowering competent corms with abscisic acid (ABA) suppressed both floral induction and flower formation, whereas some other hormones, like auxins (indole acetic acid, IAA) and gibberellic acid (GA), behaved contrarily at different developmental stages. IAA promoted flower induction, while GA suppressed it; however, GA promoted flower formation, whereas IAA suppressed it. Cytokinin (kinetin) treatment suggested its positive involvement in flower induction and flower formation. The expression analysis of floral integrator and homeotic genes suggests that ABA might suppress floral induction by suppressing the expression of the floral promoter (LFY, FT3) and promoting the expression of the floral repressor (*SVP*) gene. Additionally, ABA treatment also suppressed the expression of the floral homeotic genes responsible for flower formation. GA reduces the expression of flowering induction gene *LFY*, while IAA treatment upregulated its expression. In addition to these genes, a flowering repressor gene, *TFL1-2*, was also found to be downregulated in IAA treatment. Cytokinin promotes flowering induction by increasing the expression levels of the *LFY* gene and decreasing the *TFL1-2* gene expression. Moreover, it improved flower organogenesis by increasing the expression of floral homeotic genes. Overall, the results suggest that hormones differently regulate flowering in saffron *via* regulating floral integrator and homeotic gene expression.

## Introduction

Saffron (*Crocus sativus* L.) is a monocot that belongs to the plant family Iridaceae and grows as a perennial stemless herb in Iran, Spain, Greece, India, Italy, and Nepal. Because of its triploid chromosome makeup, this plant reproduces asexually by corm feeding and produces sterile offspring (Esmaeili et al., 2011). The most valuable component of saffron is the flower, which has six tepals, three stamens, and three stigmas. Stigma is used as a spice, coloring, and flavoring agent in agro-food and cosmetic industries (Gohari et al., 2013). Flowering in saffron is highly complex and regulated by various external and internal factors. Flowering is the most important stage in saffron plant development, which is directly related to crop yield and productivity. External factors, mainly temperature, have been suggested as a major regulator controlling flowering in saffron. Despite the temperature, other regulatory networks also control flowering, including phytohormones. Plant hormones are an important factor that helps transmit signals from inside or outside the plant and contributes to the flowering process (Conti, 2017). Considering the importance of flowering in saffron production, several studies have been conducted to understand the process, with the majority of them directed towards understanding the influence of temperature on saffron flowering but only a few on the effect of hormones (Molina et al., 2005a; Molina et al., 2005b; Renau-Morata et al., 2021; Javid et al., 2022). The influence of hormones on the flowering process in saffron is partly explored. Therefore, unravelling the hormonal network in saffron flower induction and formation is of great interest concerning its utilization for maximized flowering and yield.

Plant hormones, gibberellic acid (GA), abscisic acid (ABA), cytokinin, ethylene, and auxin, are known to regulate a series of developmental processes such as seed germination, plant growth, senescence, flowering, *etc*. There has always been a dilemma on the plant hormones’ exact role in regulating the flowering process—for example, GA has been positively correlated with flowering in several plant species like *Arabidopsis*, tobacco, radish, *etc*. (Gallego-Giraldo et al., 2007; Porri et al., 2012; Jung et al., 2020; Fukazawa et al., 2021), and contrary to many perennial fruit species, it inhibits flowering in apples, grapes, and citrus (Boss and Thomas, 2002; Garmendia et al., 2019; Zhang et al., 2019). GA has been shown to affect the different phases of flowering, from flowering induction to flower development (Mutasa-Göttgens and Hedden, 2009). Besides the model plants, the roles of GA in flowering have also been studied in some geophytes such as in *Zantedeschia*, lily, *Tulip*, *Allium sativum*, *Anemone*, *Paeonia*, *Lilium*, *Hyacinthus*, saffron, *etc*. ((Rudnicki et al., 1976; Evans et al., 1990; Ramzan et al., 2014; Hu et al., 2020; Renau-Morata et al., 2021; Yari et al., 2021). However, detail of the said mechanism is still required. In *Zantedeschia* GA promotes flower initiation and development processes, and the number of inflorescences is determined by the doses and duration of GA treatment (Brooking and Jamieson, 2002). In general, GA treatment has been shown to overcome and compensate for the low temperature requirement for flowering in the model plant *Arabidopsis* (Moon et al., 2003; King et al., 2008), including *Tulips* (Kurtar and Ayan, 2005). The contradictory role of GA in regulating flowering has also been suggested in different studies carried out on saffron (Renau-Morata et al., 2021). Their research correlating hormone signaling during flower induction and development suggested that flower initiation might be suppressed by gibberellins, while another study by Hu et al. (2020) suggested higher accumulations of GA and downregulation of GA2ox, an inhibitor of GA pathway, suggesting a positive role of GA in promoting flowering. Thus, more studies are required to suggest the exact role of GA in flowering regulation in saffron. ABA is another important plant hormone with a suggested role in flowering. Various studies using ABA mutants suggest a negative effect on flowering in *Arabidopsis* (Wang et al., 2013; Shu et al., 2016). Although the exact mode of action of ABA in regulating flowering is not known, ABA controls *Flowering Locus T* (*FT*) gene transcription *via GIGANTEA* (*GI*), *CONSTANS* (*CO*), and *SUPPRESSOR OF OVEREXPRESSION OF CONSTANS 1* (*SOC1*) expression (Riboni et al., 2016; Martignago et al., 2020). A negative role of ABA on flowering induction has been suggested in saffron (Hu et al., 2020; Renau-Morata et al., 2021).

Auxins are considered as a key regulator for flower development and floral organ patterning (Nemhauser et al., 1998; Aloni et al., 2006; Cheng and Zhao, 2007). Auxins can affect floral initiation in multiple ways by either promoting floral organ primordia genes or by inhibiting the pluripotency of stem cells. During floral initiation, auxins are shown to regulate the expression of *LEAFY* (*LFY*) and *APETALA 1* (*AP1*) genes in *Arabidopsis* (Yamaguchi et al., 2013; Yamaguchi et al., 2016), while at a later stage during flower organ development auxin (ARF3) was found to regulate the expression of *APETALA2* and *AGAMOUS* genes (Liu et al., 2014). In addition to auxins, cytokinins have been suggested to have a role in promoting flowering induction in many plant species—such as in *Arabidopsis*, BAP promotes flowering *via* the activation of FT paralog TSF (TWIN SISTER OF FT) (D'Aloia et al., 2011; Han et al., 2014); in rose, cytokinin promotes flowering production (Zieslin et al., 1985); in strawberry, cytokinin is also involved in flowering (Eshghi and Tafazoli, 2007); and in *Iris*, zeatin and isopentenyl-adenine play an important role in flower bud blasting (Vonk et al., 1986). The cytokinin pathway also mediates *APETALA1* function in the establishment of floral meristems in *Arabidopsis* (Han et al., 2014). Different auxin and cytokinin ratios are responsible for stamen and ovule development in *Hyacinthus* from perianth explant (Lu et al., 1988). Plant hormones altogether have been suggested to have a species-specific and distinct role during different flower developmental stages.

**Figure 1:**
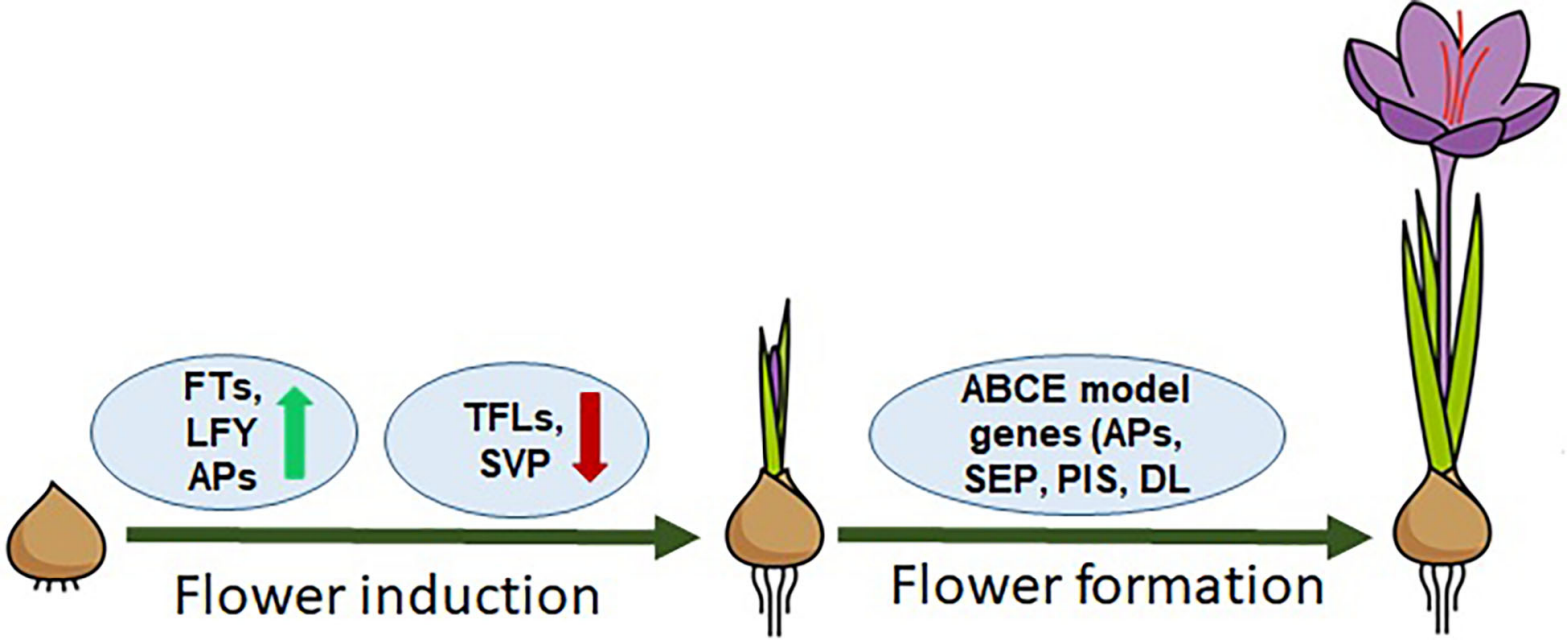
Model representing the flowering regulation in saffron. FT, LFY genes are flower inducers; TFLs, SVP are flowering inhibitors; ABCE model genes: APs, SEPs, PISs, and DLs are homeotic genes/flower organogenesis.

The flowering process in saffron, similar to other plant species, can be divided into two main stages, *i*.*e*., (1) floral induction/initiation and (2) floral organ development. These two distinct developmental stages have different sets of genes involved and categorized as floral integrators and floral homeotic genes (**Figure 1**). Floral integrator genes comprising *FT, LFY, SVP* and *TFL* genes. *FT* and *LFY* regulate positively while *TFL* and *SVP* negatively regulate the flowering induction. (Tsaftaris et al., 2007; Kalivas et al., 2007a; Kalivas et al., 2007b; Tsaftaris et al., 2010; Tsaftaris et al., 2011; Tsaftaris et al., 2012; Tsaftaris et al., 2013; Kalia et al., 2022), while floral homeotic genes consist of ABCE model genes such as *APETALA*, *PISTILLATA*, *SEPALLATA*, and *DROOPING LEAF*-like genes that are involved in floral organ development and patterning (Renau-Morata et al., 2021). The role of a few of these genes has been studied—for example, a lot still remain undeciphered concerning flowering regulation in saffron. By analyzing *PEBP* genes, floral integrator genes in saffron have been identified, but their hormonal regulation remain unexplored (Tsaftaris et al., 2013; Kalia et al., 2022). The spatiotemporal expression profiling of FT genes identified from saffron has suggested different roles during flowering and vegetative growth. *CsatFT3* gene has been suggested in temperature and sugar mediated regulation of flowering in saffron (Tsaftaris et al., 2013; Kalia et al., 2022; Jose-Santhi et al., 2023). A *SVP* homolog gene, which is a negative regulator of flowering, was shown to be expressed only in the vegetative buds of small-sized corms, which are not competent to flower (Haghighi et al., 2020). This finding indicates that, similar with other plants, SVP-like genes might be involved in regulating *FT* gene expression in saffron. However, more research is required to provide a detailed analysis. Similarly, floral homeotic genes have been identified in saffron, but studies are confined only to cloning and limited expression analysis. The plausible roles of these genes were based on sequence similarities and expression analysis (Tsaftaris et al., 2007; Kalivas et al., 2007a; Tsaftaris et al., 2011). Additionally, the major limitation in studying the hormonal regulation of flowering in saffron is that, when applied exogenously, their effect does not last longer and the whole flowering process can take months and is ineffective for observing long-term effects. In that case, it is very difficult to predict that it is the cause or the consequence that is seen after a long interval of hormone application.

Thus, in the present study, to define specific function of a hormone in regulating a distinct developmental stage, we have used two stages for hormone application. The first exogenous hormone application was done at the dormant stage, and it was followed for their ability to induce the process. Secondly, to understand their role in flower formation i.e. development of flowers, corms with pre induced flowering process were used. After hormone treatment, the morphological, histological, and transcriptional changes during different developmental stages were studied. The study suggests that hormones differentially affect flower induction and formation in saffron *via* regulation of floral integrator and Homeotic genes expression.

## Materials and methods

### Plant material

Saffron (*Crocus sativus* L.) corms grown in the field of CSIR-IHBT, Palampur, Himachal Pradesh, India, were used for the study. The corms were planted in October 2020 and harvested in April 2021. After harvesting, the corms were air-dried and sorted according to weight for the experiments. Corms with a weight range of 10–12 g that are considered competent to flower were used for the experimentation. In total 20 corms per treatment were used for each developmental stages. Different tissues of the saffron plant—namely, leaves, roots, corms, and flower tissues (stigma, stamens, and tepals)—were also collected separately for tissue-specific expression of genes.

### Hormonal treatment

To understand the role of hormones involved in flowering induction, corms 10–12 g in size were treated with different hormones. Briefly, a hormonal solution of IAA (0.5 mg/l or 2.85 μm), ABA (0.5 mg/l or 1.89 μm), GA (0.5 mg/l or 1.44 μm), and kinetin (0.5 mg/l or 2.32 μm) was prepared in distilled water, and the corms were dipped in solution and then infiltrated for 2 h under vacuum. The control sample was dipped only with distilled water for the same span of time. After treatment, the corms were wiped with tissue, dried, and then stored at 25°C for floral induction in the dark for 90 days. Samples were collected for morphological and gene expression analysis from apical buds after 45 days (stage 1) and 90 days (stage 2) of treatment. Three biological replicates for each treatment were used for the study. The samples were harvested, frozen in liquid nitrogen, and stored at -80°C until further use.

To study the role of hormones in flower formation, flowering competent corms (10–12 g) that were stored at 25°C for 3 months in a growth chamber (June–August) and which already have floral initiation in them were used for the experiment. In early September, after exogenous hormone treatments (IAA, ABA, GA, and kinetin), corms were stored at 15/20°C during the night and day, respectively, with 8/16-h light/dark cycle for flower emergence in the growth chamber. Samples were collected at 45 days’ interval in mid-October (stage III) and at the end of November (stage IV) when flowers emerged and were visible.

### Histochemical and morphological analysis

Apical bud tissue at 45 days of the developmental stage of large corms were collected and fixed quickly in 4% paraformaldehyde solution. The fixed samples were dehydrated with ethanol series (10%-100%). Samples were stained with 0.05% eosin and replaced by Histo-Clear gradually. Then, the samples were embedded in paraffin and sliced to a thickness of 7–10 µm with Thermo Scientific HM 355S automatic rotary microtome. The sliced samples were photographed under Leica DM2000 optical microscope (Javelle et al., 2011).

### Gene selection for the study

The genomic resource (genome sequences) of saffron is not yet available, making it difficult to mine genes involved in the process. In order to study the genes involved in flowering regulation, we selected genes which were previously reported either in saffron or other plants. Briefly, floral integrator genes that comprise *FTs*, *TFL1s*, *LFY*, and floral repressor *SVP* along with floral homeotic genes (*APATELLA*, *PISTILLATA*, *SEPATELLA*, *DROOPING LEAF*, *etc*.) were obtained from in-house and online database and used for the study. The list of genes studied, their source, and the references used in the present work have been listed in **Supplementary Table S1**.

#### RNA extraction and cDNA preparation

Total RNA was extracted from apical tissues using Spectrum Plant Total RNA kit (Sigma-Aldrich) according to manufacturer’s instructions. Portions of RNA at 10 μg were treated with RNase-free DNase I (Thermo Fisher), and portions 2 μg were then utilized in cDNA preparation. The cDNA was prepared using a verso cDNA synthesis kit (Thermo Fisher) as per the manufacturer’s instruction. Expression analysis was done by using RT-PCR.

#### RT-PCR and amplification conditions

Real-time PCR (RT-PCR) was done to quantify the expression of genes using gene-specific primers (**Supplementary Table S2**). The reactions were performed using three biological replicates and three technical replicates on Applied Biosystems real-time PCR machine. The analyzed real-time reaction data was the mean of biological and technical triplicates. The PCR conditions were as follows: 2 min at 50°C, 95°C for 3 min, followed by 40 cycles of 15 s at 95°C, 15 s at 55°C, and 45 s at 72°C. Tubulin was used as the internal control.

### Statistical analysis

Tukey–Kramer multiple-comparison test by GraphPad software (significance level of *P* < 0.05; GraphPad URL: https://www.graphpad.com) and one-way ANOVA test (significance level of *P* < 0.05) were used to analyze the real-time PCR and corm morphological data, respectively.

## Results

### Morphological changes in the apical bud during flowering

To identify the morphological changes and development of different organs during the flowering process, the apical buds were monitored for 6 months (May–November), and apical bud samples were collected and analyzed after every 45 days from the start of the experiment. The morphological studies suggest that flower induction occurred in the first 45 days (stage 1), whereas the stamen and stigma start to develop between 45 and 90 days (stage 2). Stage 3 is marked with elongation in the stamen and stigma, and tepals development was observed between stage 3 and stage 4. A complete flower with all the organs developed was seen at stage 4 (**Supplementary Figure S1**).

### Morphological changes in response to different hormones during flower induction

**Figure 2:**
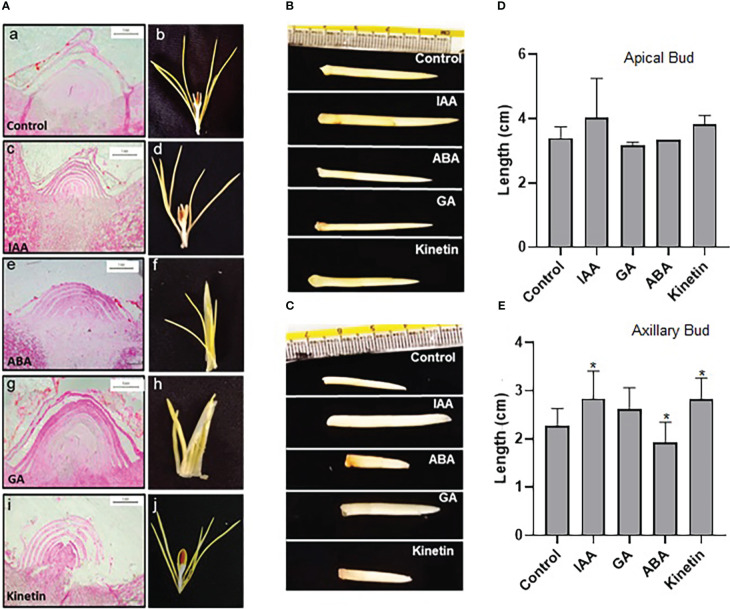
Morphological characteristics of saffron apical bud during flower induction stages after the hormonal treatments. **(A)** Cross-section images of the apical bud after 45 and 90 days of hormonal treatments, representing stage I and stage II, respectively: a, b—control (mock treatment); c, d—IAA-treated; e, f—ABA-treated; g, h—GA-treated; and i, j—kinetin-treated in stage I and stage II, respectively. **(B, C)** Representative pictures of the apical bud and axillary bud, respectively, in stage II of treatments. **(D)** Graphical representation of apical bud length. **(E)** Graphical representation of axillary bud length in stage II. The data presented is the mean of three biological replicates. Error bars represent standard error. *P < 0.05, with respect to control (Student’s *t*-test).

To study the effect of hormones on flower induction in saffron, dormant corms were treated with different hormones (GA, ABA, IAA, and kinetin) and were analyzed for floral induction process. After 45 days (stage 1), the apical meristem was fixed and used for microscopic analysis after sectioning. The morphological studies suggest that GA and ABA inhibited flower induction as seen in the longitudinal cross-section of the apical bud (small and semi-conical in shape, undifferentiated apical bud meristem tissues) (**Figure 2A**). Corroborating with the results seen after 45 days in ABA and GA treated samples, no floral organ formation can be seen in them after 90 days (**Figure 2A**). However, in the control, IAA- and kinetin-treated apical bud floral induction was prominently visible (differentiated apical bud meristem tissues), which is further confirmed by apical bud images at 90 days (stage 2) after the respective treatment that shows the development of the stamen and stigma (**Figure 2A**). The effect of different hormone treatments was also visible on apical and axillary bud length. IAA and kinetin promoted apical bud growth, which is reflected as increased apical and axillary bud length, whereas a significant reduction in apical and axillary bud length is observed in ABA and GA treated samples (**Figures 2D, E**).

### Expression analysis of floral integrator and homeotic genes during flowering in saffron

**Figure 3:**
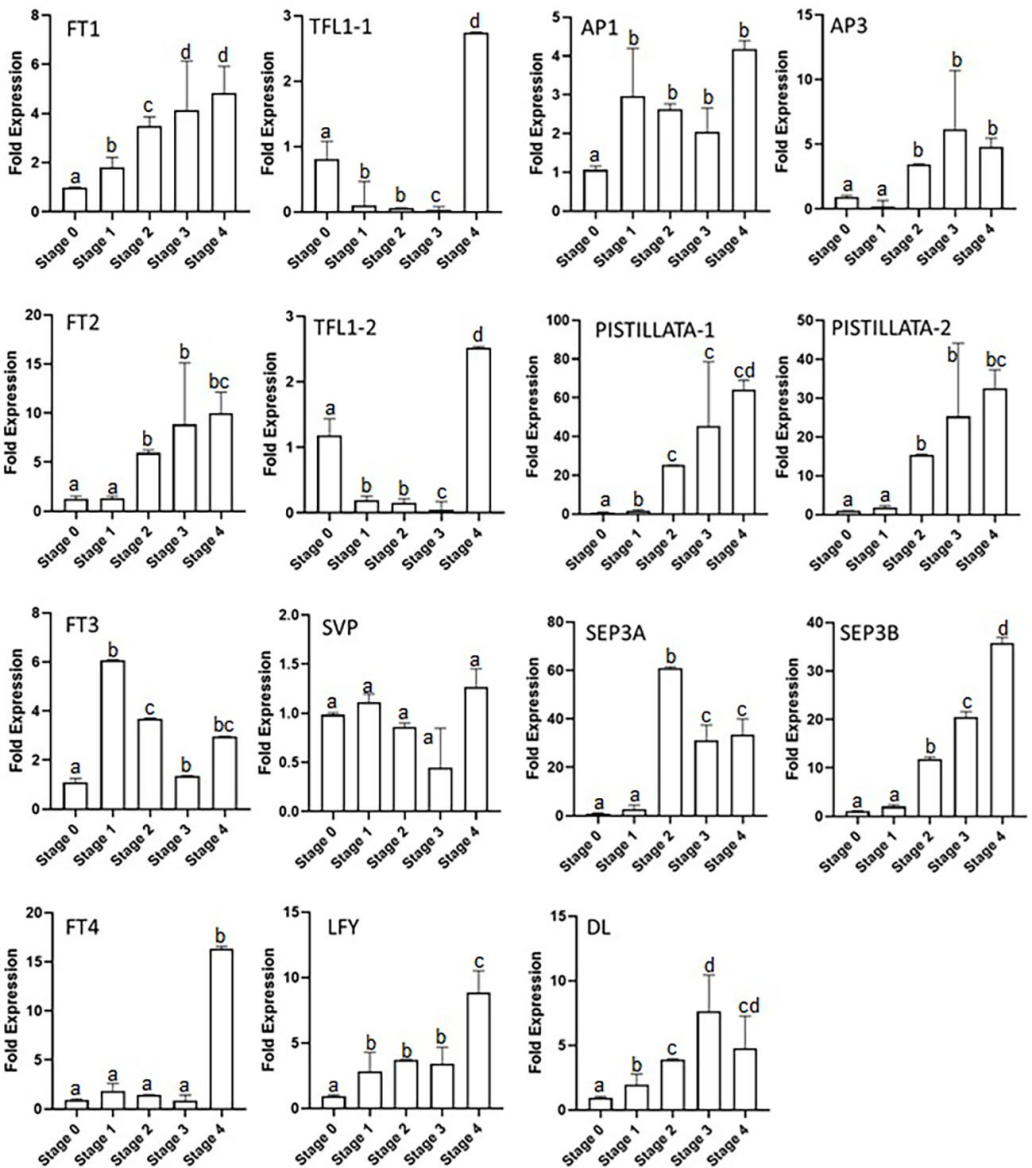
Expression profiling of floral integrator and homeotic genes at different developmental stages of saffron flowering. Stages 0–4 represent different developmental stages during saffron flowering. Stage 0, dormant corms; stage 1, flower induction stage; stage 2, stamen and stigma formation; stage 3, stamen and stigma development and elongation; and stage 4, tepal development. Error bars represent ± SD of three biological replicates. Stage I morphologically represents the flower induction stage, and stage II represents the stamen and stigma formation stage. Letters (a–d) over the bars indicate significant differences at *P* < 0.05 (means followed by the same letter are not significantly different at *P* = 0.05).

The expressions of floral integrator genes (*FTs*, *TFL1s*, *LFY*, and *SVP*) were checked during the different stages of the flowering process. The results show that *CsatFT1* and *CsatFT2* gene expression gradually increased from stage 0 to stage 4 by up to three- and fivefold, respectively (**Figure 3**). The increase was observed from stage 1 and 2 in *CsatFT1* and *CsatFT2* genes, respectively, and no significant increase in expression was seen at stage 0 (flowering induction stage). However, *CsatFT3* comparatively showed a sixfold increase in stage 1, and then its expression declined in further stages. The *CsatFT4* gene transcript levels remained low throughout the stages and rapidly increased by up to ~15 folds in stage 4. The *CsatTFL1-1* and *CsatTFL1-2* genes showed a similar expression pattern (**Figure 3**). The transcript of TFLs was higher at stage 0 but reduced rapidly in stage 1 and remained low throughout all the stages except at stage 4, where their expression increased again by more than approximately twofold. *CsatLFY* gene expression was observed from stage 0, and it gradually increased at further stages. The expression of *CsatSVP* gene was not found to be significant at the early developmental stages but declined slightly at stage 3 and stage 4 (**Figure 3**).

The homeotic genes showed a different expression in the developmental stages of saffron, such as the *CsatAP3* expression which started to increase from stage 2, had its highest expression by up to approximately fivefold in stage 3, and further showed a small reduction of its transcript levels in stage 4. *CsatPISTILLATA-1* and *CsatPISTILLATA-2* showed the same expression patterns (**Figure 3**). The *CsatPISTILLATA-1* and *CsatPISTILLATA-2* transcripts started to appear from stage 2 and stayed highest by approximately 60- and 30-fold, respectively, in stage 4. The *CsatSEP3A* and *CsatSEP3B* genes had different expression patterns. The *CsatSEP3A* gene expression was highest in stage 2 (approximately 60-fold), and the transcript levels were rapidly decreased by 60%–70% in stage 3 and stage 4. The *CsatSEP3B* transcripts were also accumulated from stage 2 and went to its highest by ~30-fold in stage 4 (**Figure 3**). Another gene, *DL*, showed a gradual increase in expression pattern from stage 1 to stage 3 (approximately sevenfold), while the transcript levels were decreased by 40%–50% in stage 4. The *AP1* gene also started to accumulate from stage 1 and declined slightly in stage 3 and stage 4 (**Figure 3**).

### Tissue-specific expression profiling of floral integrator and homeotic genes

Floral integrator and homeotic genes show tissue-specific expression in plants, suggesting their role in different plant and organ developmental processes. Our previous studies have provided the tissue-specific expression of *FT* and *TFL* genes in saffron (Kalia et al., 2022), but it remains unknown for floral homeotic genes and other genes such as *SVP* and *LFY*. Thus, we examined the expression of these genes in different tissues. The expression patterns found that *CsatDL* (dropping leaf) gene was highly and specifically expressed in the stigma tissue (**Supplementary Figure S2**). The *CsatPISTILLATA-2*, *CsatSEP3A*, *CsatSEP3B*, and *CsatAP3* genes were expressed in stamen, stigma, and tepals tissues, whereas *CsatPISTILLATA-1* expression was confined to tepals and stigma tissues (**Supplementary Figure S3**). However, *SVP* expressions were only observed in leaf and corm tissues of saffron. The *CsatLFY* gene was expressed in all the tissues studied, with a higher expression in leaf and corm tissues. Similar to *CsatLFY*, *CsatAP1* expression was also found in most of the tissues except in the roots.

### Effect of hormone treatment on the expression of floral integrator genes during floral induction

**Figure 4:**
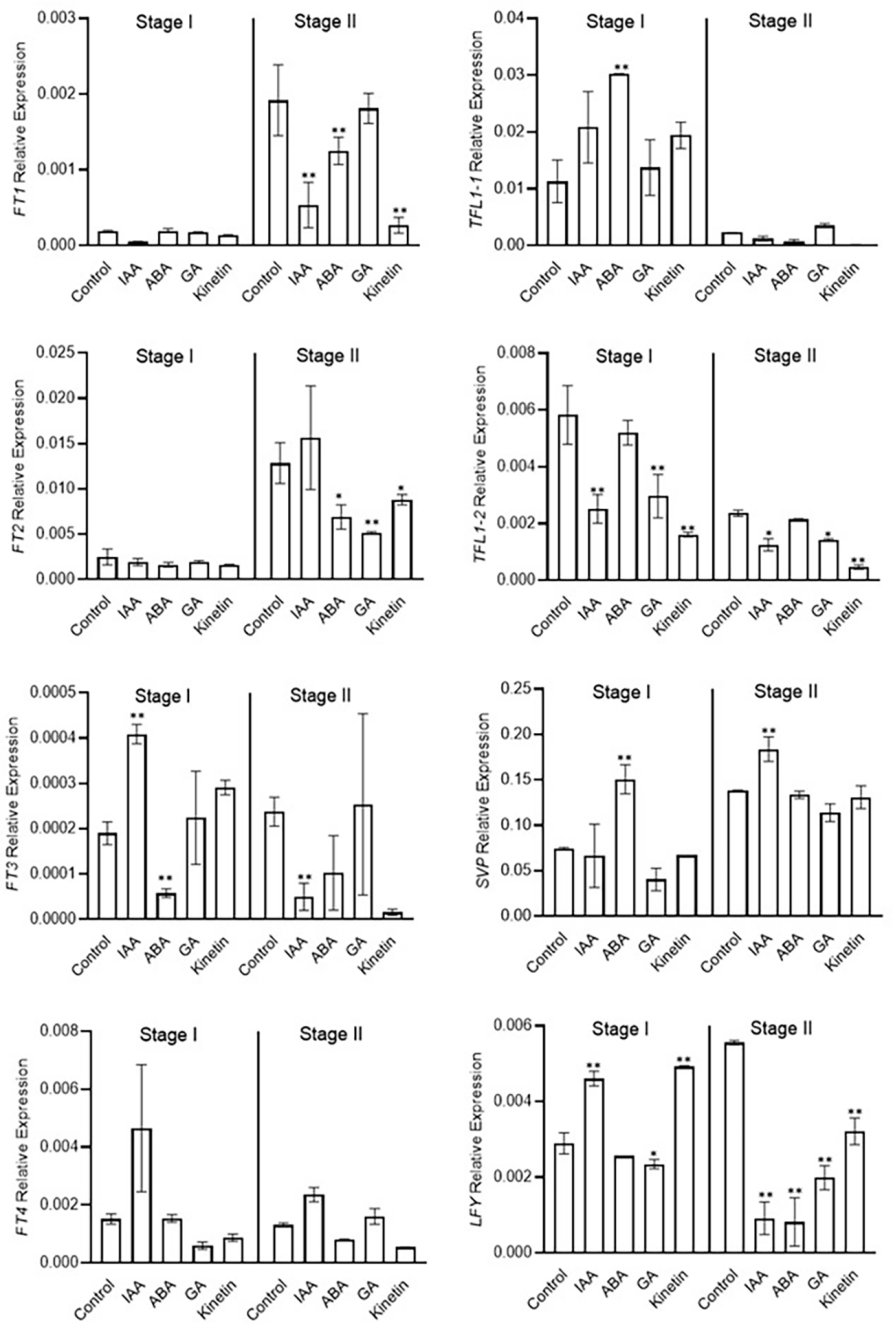
Expression profiling of genes involved in flowering induction quantified by real-time PCR in response to different hormones. Total RNA was isolated from the apical bud of corms treated with different hormones at 45 days (stage I) and 90 days (stage II) of treatments. Reactions from three separate pools of apical bud RNA samples were run in triplicates with tubulin as the internal control for normalization. Error bars represent ± SD of three biological replicates. Stage I morphologically represents the flower induction stage, and stage II represents the stamen and stigma formation stage. Data represented is mean of three biological replicates. Error bars represent standard error (SE), * indicates *P* < 0.05, ** indicates *P* < 0.01, with respect to control (Student’s t-test).

Morphological studies identified stage 1 as the flower induction stage in saffron flowering. Hormone treatments also differently affected them. We next examined the expression of previously identified/suggested floral integrator genes to establish a correlation between the effect of hormone on flower induction and organ formation *via* their transcriptional regulation. *FT* and *LFY* genes are positive regulators of floral induction. In our study ABA and GA treatments negatively co-related with flowering induction (**Figure 2A**). ABA and GA significantly reduced the expression of *LFY* gene in stage 2 (**Figure 4**). Moreover, ABA reduced the the expression of *FT3* gene in stage 1 (**Figure 4**). No significant effect of ABA and GA was observed on *CsatFT1*, *CsatFT2*, and *CsatFT4* expression compared with the control. Moreover, ABA significantly induced the expression of *CsatTFL1-1* and *CsatSVP* genes that are considered as floral repressors at floral induction stage 1 (**Figure 4**). Contrary to this, IAA and kinetin, which promoted flower induction, also promoted the expression of *CsatFT3* and *CsatLFY* genes that are known to be involved in floral induction. At stage 2, most of the positive floral integrator genes were downregulated significantly, while floral repressors were upregulated during this stage in comparison with the control (**Figure 4**).

### Effect of different hormones on the expression of floral homeotic genes at the early stage of flower organ differentiation

**Figure 5:**
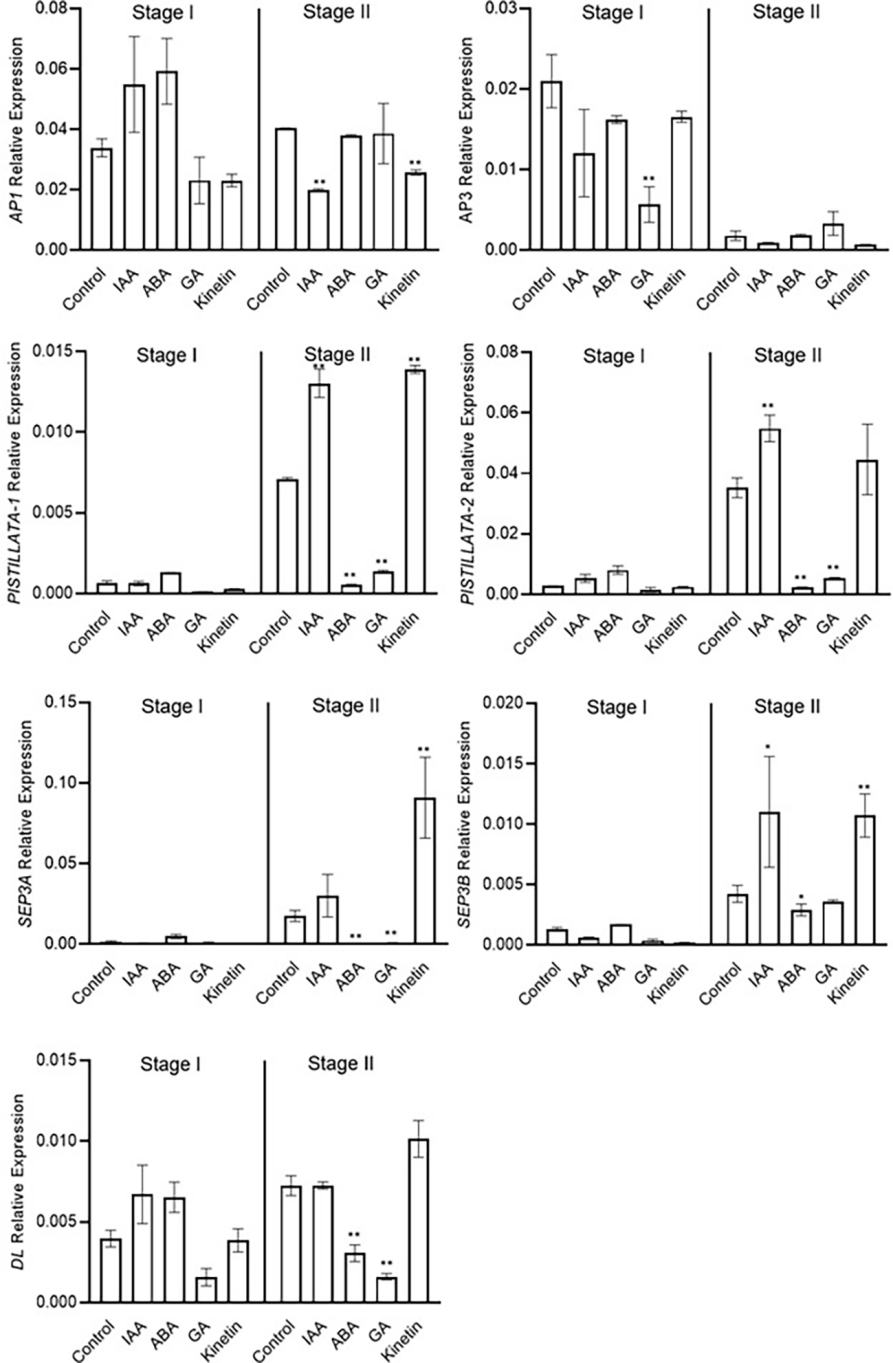
Expression profiling of genes involved in flower formation quantified by real-time PCR in response to different hormones. Total RNA was isolated from the apical bud of corms treated with different hormones at 45 days (stage I) and 90 days (stage II) of treatments. Reactions from three separate pools of root RNA samples were run in triplicates with tubulin as the internal control for normalization. Error bars represent ± SD of three biological replicates. Stage I morphologically represents the flower induction stage, and stage II represents the stamen and stigma formation stage. Data represented is mean of three biological replicates. Error bars represent standard error (SE), * indicates *P* < 0.05, ** indicates *P* < 0.01, with respect to control (Student’s t-test).

Stage 2 was marked as the stamen and stigma differentiation stage (**Figure 2**). To identify the role of conserved floral homeotic genes, also known as ABCE model genes, and their regulation *via* different hormones, we studied the expression of these genes during stage 1 and stage 2 of flowering. The expression of the studied genes was not significantly changed during stage 1, and majority of these genes were expressed at low levels except for the *CsatAP1* and *CsatAP3* genes (**Figure 5**). The AP3 gene was significantly downregulated in GA treated samples. A significant reduction in the gene expression of *CsatPISTILLATA-1* and *CsatPISTILLATA*-2 (involved in stamen and tepals formation) was observed in ABA and GA treated samples, whereas upregulation was seen in IAA and kinetin treated samples, respectively, at stage 2 (**Figure 5**). Similarly, members of the *CsatSEPTELLA* (*SEP3A* and *SEPB*) genes were also downregulated in ABA and GA treated samples and upregulated in IAA and kinetin treated samples at stage 2. The *dropping leaf* (*DL*)-like gene also showed a significant downregulation in expression compared with the control in ABA and GA treated samples (**Figure 5**). Overall, the results suggest that hormones that negatively affect flower induction and differentiation suppressed the expression of floral homeotic genes involved in stamen and stigma development at the early stages of flower organ differentiation.

### Hormonal effect on flower formation

**Figure 6:**
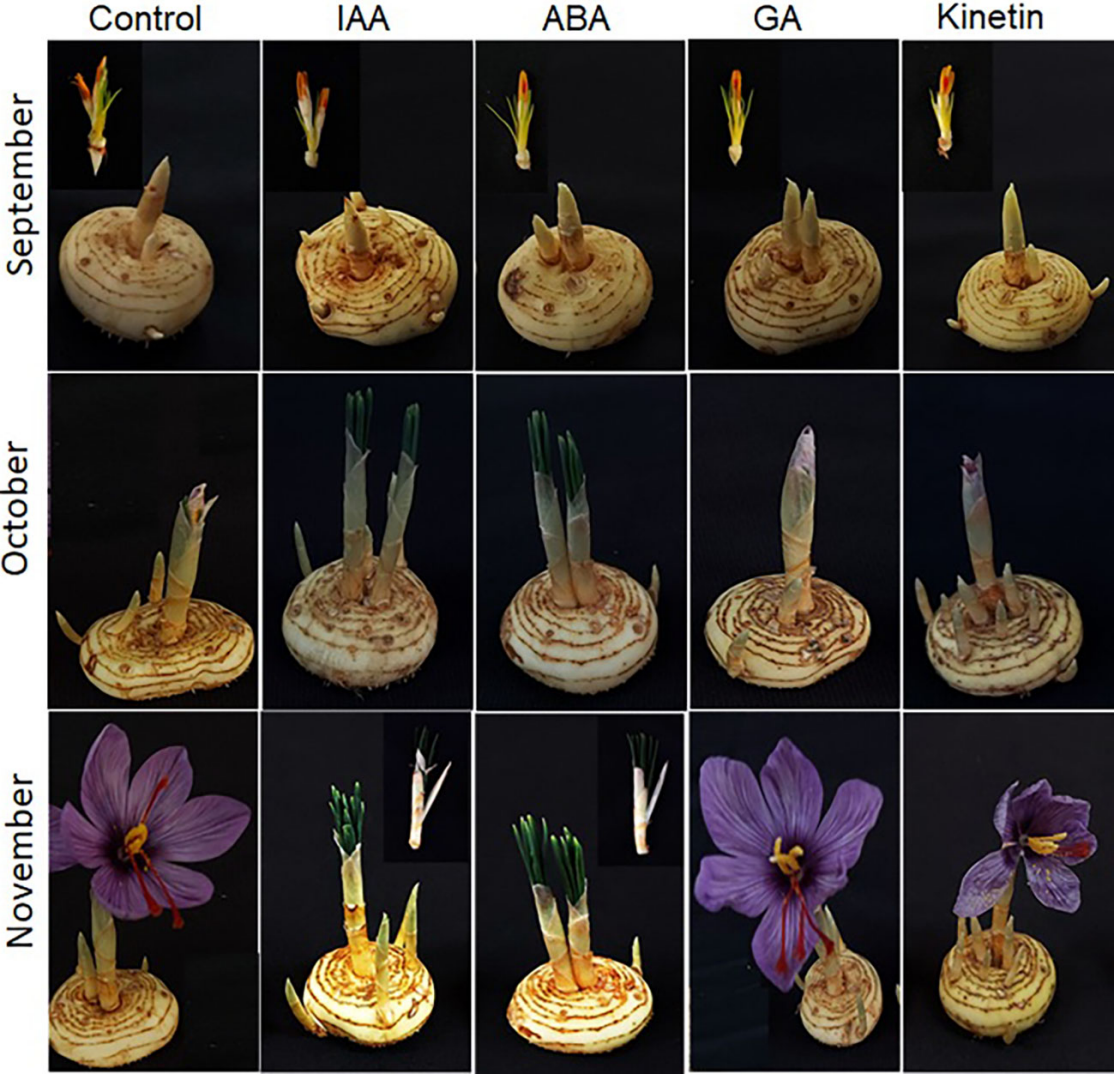
Morphological changes during flower formation in saffron after hormonal treatments. Corms already floral-initiated in early September were treated with different hormones and sampled after 45 days (mid-October) and 90 days (end of November).

To further investigate the roles of hormones in the flower formation of saffron, hormone treatments were performed in corms that have already induced flowering. After treatment, the corms were observed for flower development mainly flower formation. The results suggest that ABA just like in floral induction negatively affected flower formation. The corms that were treated with ABA showed flower atrophy, and the already initiated flower did not develop further, but the corm produced healthy leaves although with damage to the corms (**Figure 6**). Similar results were observed in IAA treated corms (flower atrophy with normal leaf development). On the other hand, GA and kinetin treatment accelerated flower formation compared with the control (**Figure 6**). The flowers in GA and kinetin treated corms showed no major difference in flower formation compared with the control. Interestingly, IAA, which promoted flower induction, suppressed flower formation, whereas GA that inhibited flower induction showed no significant effect on flower formation (**Figure 6**).

### Differential regulation of flower development genes (ABCE model) in response to different hormones

**Figure 7:**
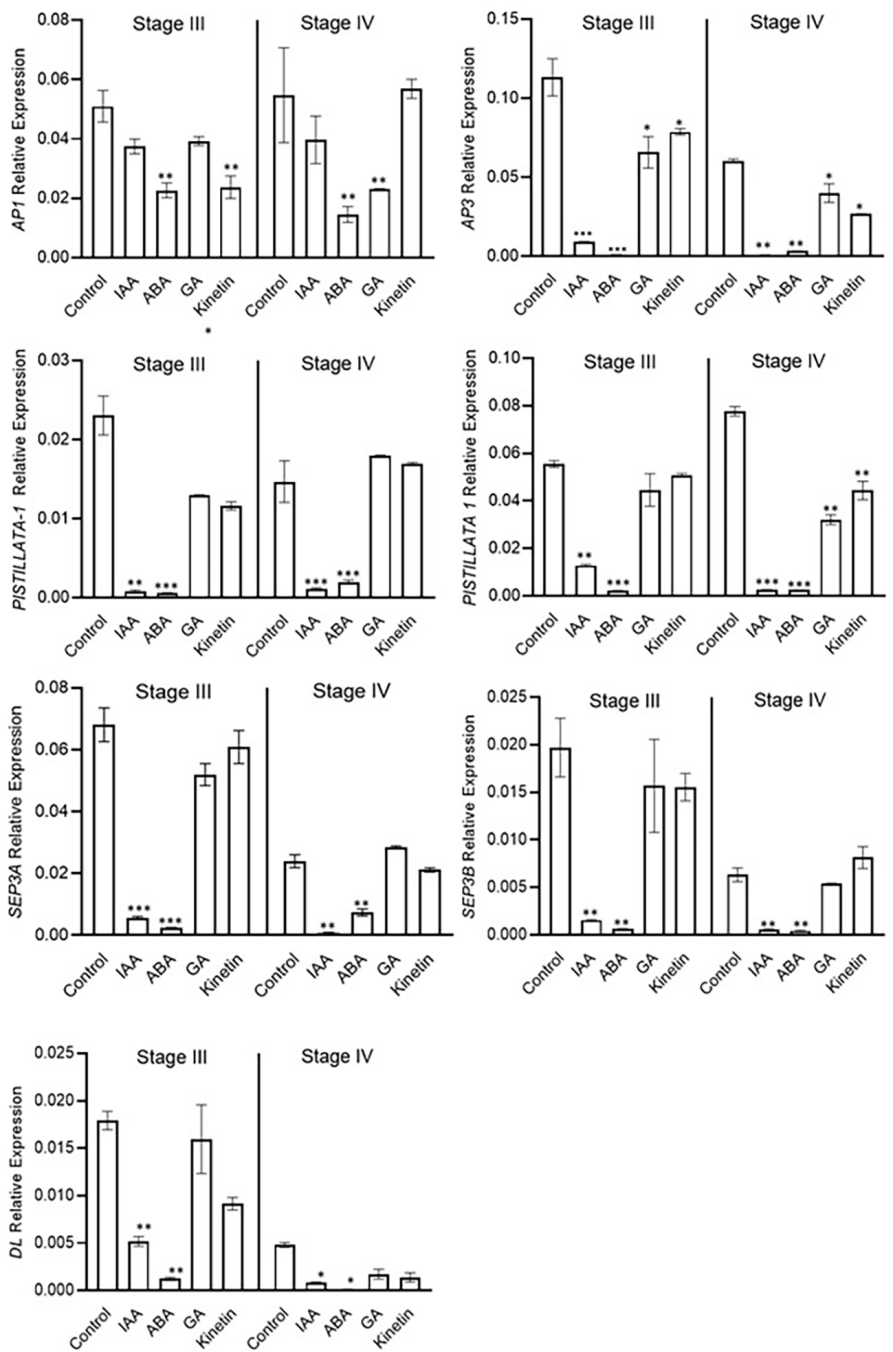
Expression profiling of genes involved in flower formation after the hormone treatment. Corms already initiated flowering were used for the treatment. The apical bud samples were collected after 45 and 90 days after the hormonal treatment. Error bars represent ± SD of three biological replicates. Stage III morphologically represents the stamen and stigma elongation stage, and stage IV represents the tepal formation stage. Data represented is mean of three biological replicates. Error bars represent standard error (SE), * indicates *P* < 0.05, ** indicates *P* < 0.01, *** indicates *P* < 0.001 with respect to control (Student’s t-test).

The expression analysis of flower formation genes was carried out at stage 3 and stage 4 of saffron flowering after different hormone treatments. ABA and IAA negatively affected flower formation and also downregulated the expression of ABCE model genes (**Figure 7**). The expression of *CsatAP3*, *CsatPISTILATTA-1*, *CsatPISTILATTA-*2, *CsatSEP3A*, *CsatSEP3B*, and *CsatDL*-like genes were significantly downregulated in stage 3 and stage 4 in ABA and IAA treated corms (**Figure 7**). The results correlated with the flower atrophy phenotype observed in corms treated with these hormones. There was no significant upregulation of any of the genes studied in GA and kinetin treated corms in comparison with the control. Moreover, the expression of most of these genes also decreased in GA and kinetin treated corms such as *CsatPISTILLATA2*, *CsatAP3*, and *CsatDL* (**Figure 7**). The expression of floral homeotic genes suggests that hormones regulate their expression in determining floral organ formation.

## Discussion

Flowering is an important development process which determines plant productivity. The flowering process in plants is controlled by various environmental factors such as light, photoperiod, vernalization, and hormones (Freytes et al., 2021). Among them, plant hormones are one of the important factors that transmit signals from inside or outside the plant and play crucial roles in regulating the flowering process (Khodorova and Boitel-Conti, 2013; Conti, 2017). Hormones are present in minute quantities, and determining their endogenous content and its correlation in regulating a process is difficult. Hence, exogenous treatments are commonly used to study their effect on several plant development processes. Different hormones have been suggested to play a role during the process, but their distinct role in regulating the different developmental stages of flowering in saffron has not been elucidated. As the saffron flower develops, it goes through two distinct stages: flower induction and flower development or organogenesis. This study found that, in addition to temperature, these developmental stages are differentially regulated by hormones.

The spatiotemporal expression profiling of floral integrator genes has been previously described in saffron (Kalia et al., 2022). Tissue- and time-specific regulation of floral homeotic gene expression ensures proper and precise floral organ development. The studied floral homeotic genes showed tissue- and stage-specific expression patterns, suggesting that they are involved in specific organ development processes similar to other plants (Liu and Mara, 2010). Similarly in other plants and in saffron, the *PISTILLATA* and *SEPATALLA* genes showed stamen-, stigma-, and tepal-specific expression, suggesting that they are involved in saffron flower organ development (Kalivas et al., 2007b; Tsaftaris et al., 2011). The expression of these genes correlated with specific stages when the stigma, stamen, and tepals are formed. Recently, a study carried out by Renau-Morata et al. (2021) reported for the first time *DL*-like genes and their involvement in flower formation in saffron but which lacked tissue-specific expression. Our results showed stigma-specific expression of *DL* and its expression during carpel (stigma, style, and ovule) development and suggest its involvement in the process. Our results are in accordance with the partial results shown by Kalivas et al. (2007a); Tsaftaris et al. (2011); Renau-Morata et al. (2021), and Tsaftaris et al. (2010).

### Gibberellic acid has a distinct role during flowering

In our study, we found that hormones differentially affect the induction and formation of flowers in saffron. GA is the class of hormones which is best documented in the flowering process of *Arabidopsis*. It enhances the flowering process in *Arabidopsis* plant (Mutasa-Göttgens and Hedden, 2009), but, on the other hand, restricts flowering in several perennial species (Boss and Thomas, 2002; Garmendia et al., 2019). GA regulates flowering in *Arabidopsis* by promoting the *LFY* and *FT* genes expression (Moon et al., 2003; King et al., 2008). This study has found that GA regulates the flower induction and flower development processes of saffron in distinct ways. GA represses the flower initiation process and promoted the flower development process in saffron. Our results are in accordance with the findings of Renau-Morata et al. (2021), which also predicted by internal GA quantification that GA might inhibit floral induction in saffron. Contrary to this though, the study by Hu et al. (2020) suggested a positive role of GA in promoting floral induction in saffron. The results presented by Hu et al. were based only on transcript analysis, and the contradiction may be due to the different stages of floral induction. It is possible that GA might naturally inhibit floral induction, as in other perennials, and promote flower organ formation. More support to our observation in the role of GA is provided by the transcriptional analysis of floral integrator genes. We observed that GA treatment significantly downregulated the expression of *CsatLFY* that is a floral integrator gene. In addition to this, GA is known to overcome the chilling requirement that is a must for many plant species to flower (Heggie and Halliday, 2004), but in the case of saffron, ambient high temperatures are required for flowering, and low temperature treatment suppresses flowering (Molina et al., 2005b). This specific temperature requirement for flower induction might be the reason for the divergent role of GA in saffron flowering compared with other flowering plants.

### ABA inhibits flower induction and flower formation

ABA acts antagonistically to GA in various developmental processes, including flowering (Vanstraelen and Benková, 2012; Liu and Hou, 2018). It has a negative effect on flower initiation in *Arabidopsis* (Wang et al., 2013). However, ABA promotes flowering in *Litchi chinensis* (Cui et al., 2013). The suppression of flowering by ABA is shown to be mediated by effecting the expression of *FT*, *SVP*, and *FLC*-like genes (Martignago et al., 2020). The effect of ABA on geophytes, including saffron, has not been studied; rather, most of the studies are on leaf senescence. As flowering in saffron is accompanied with sprouting and dormancy release, it is very interesting to see its effects on saffron flowering. Our results also show the inhibitory role of ABA in the flower induction process in saffron and the repression of *FT3* and *LFY* gene expression. In addition to this, ABA treatment also increased the expression of flowering repressor genes *CsatTFL1-1* and *CsatSVP* in stage I of the flower initiation process. *TFL* and *SVP* genes have roles in dormancy establishment and release (Singh et al., 2018) other than flowering regulation. All homeotic genes except *CsatAP3* also showed downregulation in the initiation process. A negative role of ABA in the flower induction process in saffron is also suggested by Renau-Morata et al. (2021) based on internal hormonal content and the genes involved in ABA signaling. There are not many studies on the hormonal regulation of flowering in saffron, including ABA, although the role of ABA in regulating saffron corm dormancy has been studied (Rubio-Moraga et al., 2014).

### Cytokinin (kinetin) is a positive regulator of floral induction and formation in saffron

Cytokinins have been studied in many plants in connection with flowering with contrasting roles. Cytokinin application in rice reduced the expression of *FT1* gene and delayed the flowering time (Cho et al., 2022), whereas in *Arabidopsis* it promotes flowering *via* the transcriptional activation of the *FT* paralog TSF (D'Aloia et al., 2011). Cytokinins are also important for ovule development in *Arabidopsis* (Higuchi et al., 2004; Bencivenga et al., 2012; Galbiati et al., 2013). However, this study found that cytokinin promotes both flower initiation and the developmental process of saffron by downregulating the flowering repressor gene *CsatTFL1-2* and upregulation of flower developmental genes *CsatPISTILLATA-1*, *CsatSEP3A*, and *CsatSEP3B*. Cytokinins have been implicated in dormancy release (Subbaraj et al., 2010; Wu et al., 2019) in several geophytes by the regulation of cell proliferation and division *via* cell cyclin genes. In calla lilies, a cross-talk between cytokinin and GA regulates dormancy and flowering (Subbaraj et al., 2010). Cytokinin is also essential for *in vitro* flower development in *Panax ginseng* (Soon Lee et al., 1991).

### Auxins differently affect floral induction and formation

Auxins (IAA) are another group of well-known phytohormones which regulate various aspects of plant growth and development (William et al., 2006; Spaepen et al., 2007; Spaepen and Vanderleyden, 2011). It has been found that IAA plays a crucial role in gynoecium development of *Arabidopsis* (Nemhauser et al., 2000). Our study shows that IAA is able to initiate flowering through activating flowering induction genes *FT3* and *LFY* and suppressing the flower suppressor genes *TFL1-2*. Auxin also induces the expression of homeotic genes such as *SEP3B*, *PISTILLATA-1*, and *PISTILLATA-2*. In *Arabidopsis*, auxins regulate *LFY* expression in promoting flowering (Yamaguchi et al., 2013; Yamaguchi et al., 2016). In tulips, auxin has been identified as the main hormone involved in floral induction (Rietveld et al., 2000). However, unlike kinetin, IAA has a negative impact on the development of flower formation in saffron. Our results are in corroboration with the findings of Renau-Morata et al. (2021) where they also suggested promotion of flowering induction by auxins.

## Conclusion

**Figure 8:**
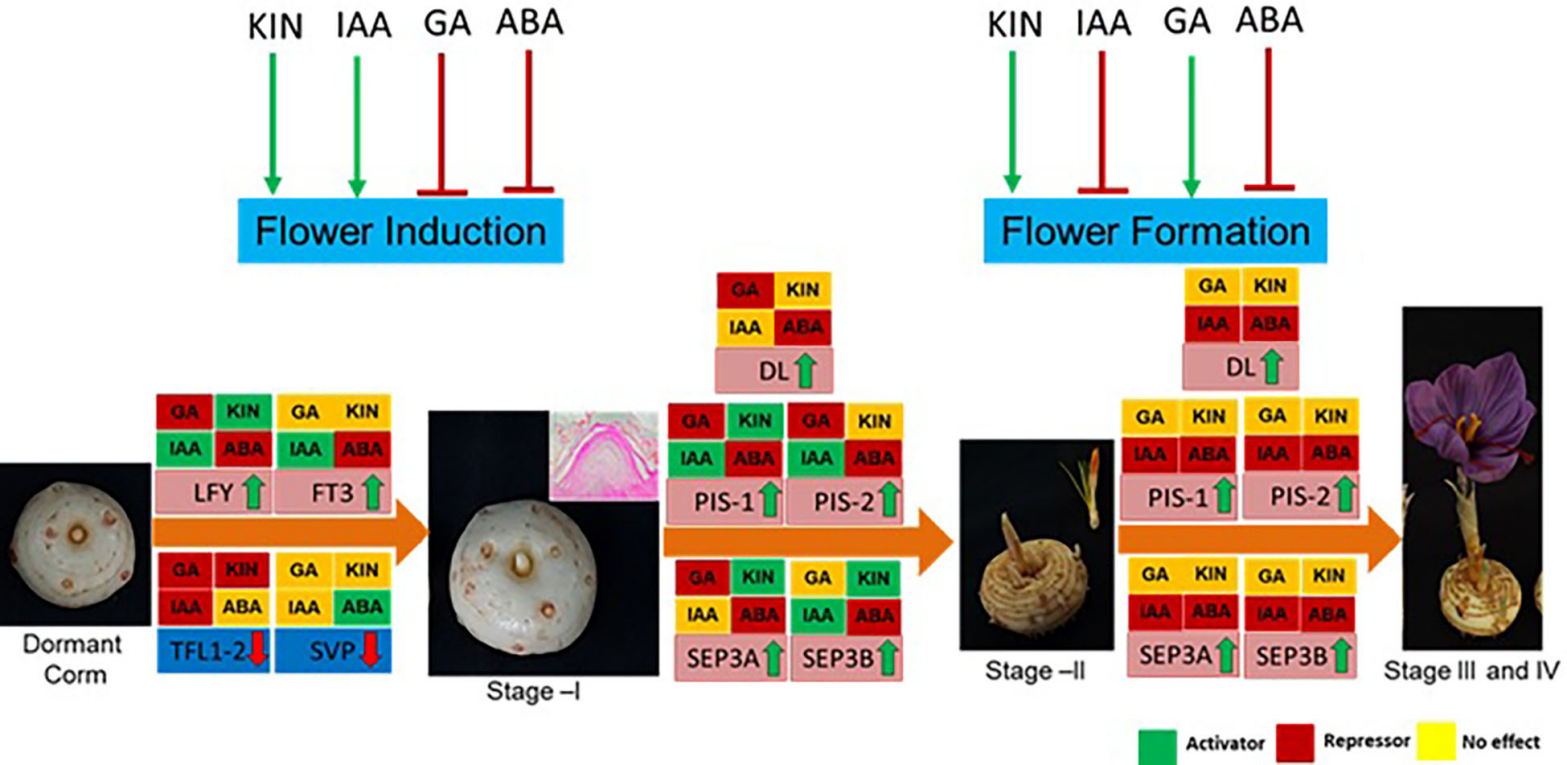
Summary of the effect of hormones on flowering process, gene regulation, and their effect at different developmental stages of saffron flowering. Briefly, ABA negatively regulates both flower induction and formation, whereas kinetin promotes both. Indole acetic acid (IAA) promotes flower induction, while gibberellic acid (GA) suppresses it. IAA inhibits flower formation, and GA promotes it. Green arrows show positive regulation, and red shows negative regulation. Hormonal effect on gene expression at different stages is marked by green, red, and yellow colors. The green color indicates induced expression levels, the red color indicates reduced expression levels, and the yellow color shows no significant changes in effect on the expression levels of the genes.

The findings of the study have been summarized in **Figure 8**. In conclusion, ABA negatively and cytokinin positively regulate both flowering induction and flower formation, whereas GA and IAA have an inverse effect on the different developmental stages. These hormones regulate the expression of genes, mainly floral integrator (*FT* and *LFY*) and repressor (*SVP* and *TFL1-2*) genes, during flower induction. Furthermore, during flower formation, they regulate the expression of floral homeotic genes (*PISTILLATA*, *SEPETALLA*, and *DL*). The findings of this study provide molecular insights into the hormonal regulation of flowering in saffron that can be utilized to alter flowering in saffron as per requirement. Additionally, the results can be utilized to induce *in vitro* flowering in saffron. Moreover, how these hormone cross-talk during the process is an area to explore that will deepen the insights of flowering regulation.

## Data availability statement

The raw data supporting the conclusions of this article will be made available by the authors without undue reservation.

## Author contributions

RS conceived the experiment idea. DS, SS, JJ-S, and DK performed the experiments and data analysis. All authors contributed to writing and reviewing the manuscript. All authors contributed to the article and approved the submitted version.
