# Supplementary material for: Hormones regulate the flowering process in saffron differently depending on the developmental stage
Source: Front Plant Sci. 2023 Mar 9;14:1107172. doi: 10.3389/fpls.2023.1107172 (PMC10034077; doi:10.3389/fpls.2023.1107172)
Supplement: Supplementary file 1 [file DataSheet_1.docx]

**Supplementary data**

**Supplementary Figure 1**

**
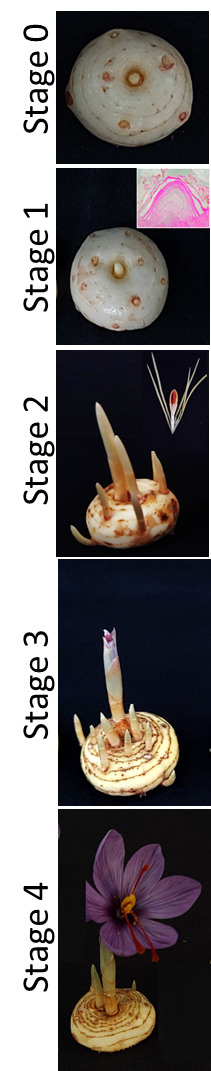
**

**Supplementary Figure 1. Representative images of different development stages in saffron.** Stage 0: dormant stage; Stage 1: Flower induction; Stage 2: stamen and stigma development; Stage 3; Stamen and stigma elongation; Stage 4: Tepal development.

**Supplementary Figure 2**

**
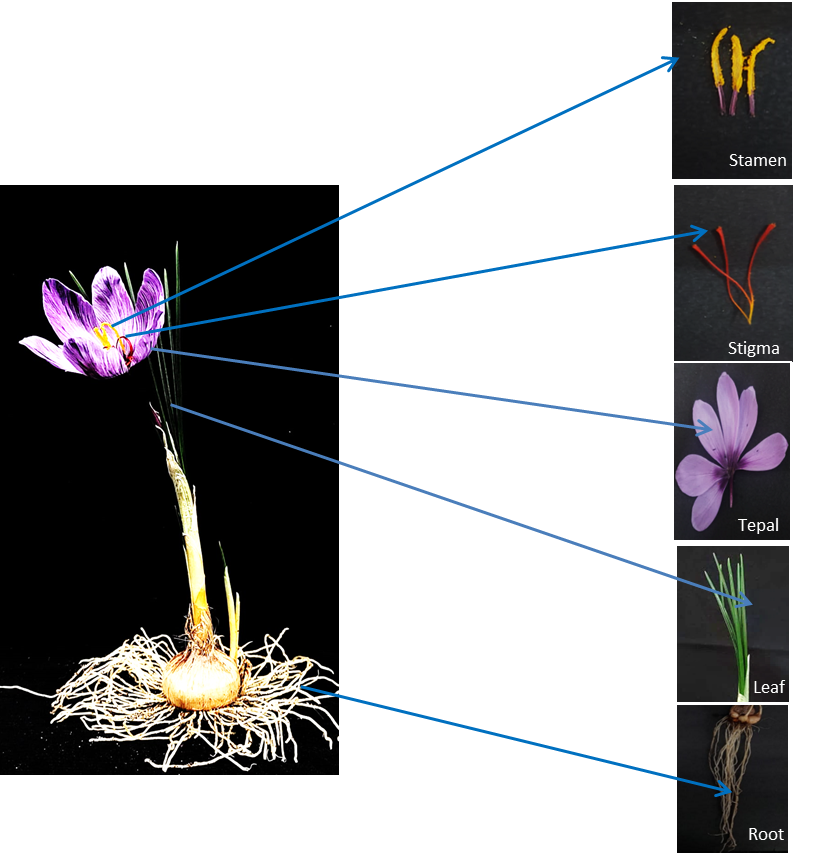
**

**Supplementary Figure 2. Representative image of different parts of saffron:** stamen, stigma, tepal, leaf, and roots.

**Supplementary Figure 3**

**
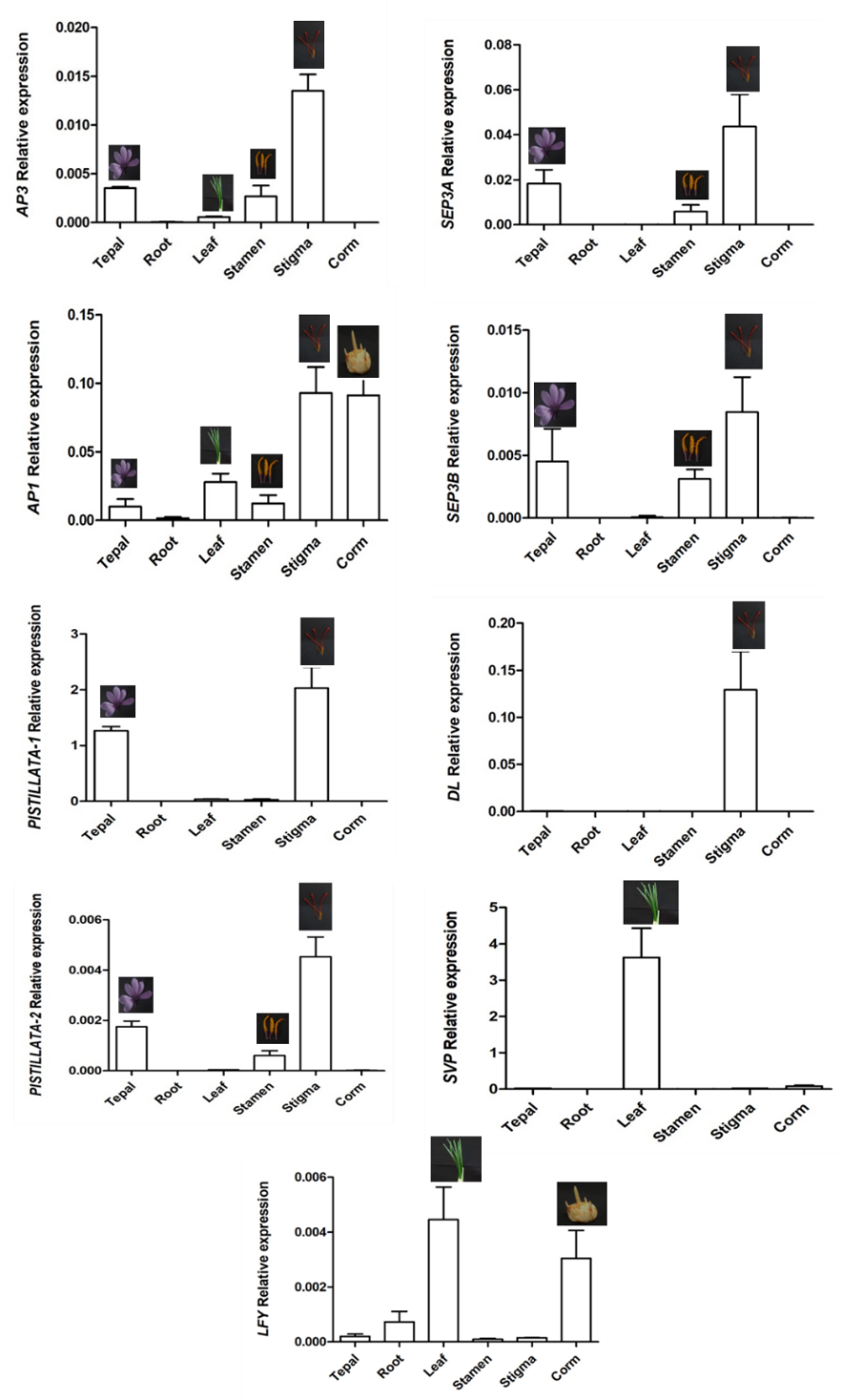
**

**Supplementary Figure 3. Tissue specific expression of saffron ABCE gene family and LFY, SVP.** Samples were collected from root, leaf, tepal, stamen, stigma and corms of same stages. Expression analysis was done by q-PCR in different tissues. Reactions were run in triplicates with *tubulin* as the internal control for normalization. Error bars represent ± SD of three biological replicates.

**Supplementary Table 1. List of genes selected for the study.**

**Supplementary Table 2. List of primers used in the study**
